# Iron Overload, Oxidative Stress and Calcium Mishandling in Cardiomyocytes: Role of the Mitochondrial Permeability Transition Pore

**DOI:** 10.3390/antiox9080758

**Published:** 2020-08-16

**Authors:** Richard Gordan, Nadezhda Fefelova, Judith K. Gwathmey, Lai-Hua Xie

**Affiliations:** Department of Cell Biology and Molecular Medicine, Rutgers University-New Jersey Medical School, Newark, NJ 07103, USA; rgordan48@gmail.com (R.G.); fefelonn@njms.rutgers.edu (N.F.); jgwathmey@gwathmey.com (J.K.G.)

**Keywords:** iron overload, oxidative stress, calcium dynamics, heart, mitochondria, arrhythmia

## Abstract

Iron (Fe) plays an essential role in many physiological processes. Hereditary hemochromatosis or frequent blood transfusions often cause iron overload (IO), which can lead to cardiomyopathy and arrhythmias; however, the underlying mechanism is not well defined. In the present study, we assess the hypothesis that IO promotes arrhythmias via reactive oxygen species (ROS) production, mitochondrial membrane potential (∆*Ψ*_m_) depolarization, and disruption of cytosolic Ca dynamics. In ventricular myocytes isolated from wild type (WT) mice, both cytosolic and mitochondrial Fe levels were elevated following perfusion with the Fe^3+^/8-hydroxyquinoline (8-HQ) complex. IO promoted mitochondrial superoxide generation (measured using MitoSOX Red) and induced the depolarization of the Δ*Ψ*_m_ (measured using tetramethylrhodamine methyl ester, TMRM) in a dose-dependent manner. IO significantly increased the rate of Ca wave (CaW) formation measured in isolated ventricular myocytes using Fluo-4. Furthermore, in ex-vivo Langendorff-perfused hearts, IO increased arrhythmia scores as evaluated by ECG recordings under programmed S1-S2 stimulation protocols. We also carried out similar experiments in cyclophilin D knockout (CypD KO) mice in which the mitochondrial permeability transition pore (mPTP) opening is impaired. While comparable cytosolic and mitochondrial Fe load, mitochondrial ROS production, and depolarization of the ∆*Ψ*_m_ were observed in ventricular myocytes isolated from both WT and CypD KO mice, the rate of CaW formation in isolated cells and the arrhythmia scores in ex-vivo hearts were significantly lower in CypD KO mice compared to those observed in WT mice under conditions of IO. The mPTP inhibitor cyclosporine A (CsA, 1 µM) also exhibited a protective effect. In conclusion, our results suggest that IO induces mitochondrial ROS generation and ∆*Ψ*_m_ depolarization, thus opening the mPTP, thereby promoting CaWs and cardiac arrhythmias. Conversely, the inhibition of mPTP ameliorates the proarrhythmic effects of IO.

## 1. Introduction

Iron overload (IO) conditions can be found in patients with hereditary hematochromatosis or in patients receiving frequent blood transfusions (e.g., for treating sickle cell disease or thalassemia). For example, patients suffering from hematochromatosis are genetically predisposed to absorb excessive dietary Fe and thus build up high plasma Fe levels. The resultant non-transferrin bound iron accumulates in organs and tissues, leading to organ damage and potentially organ failure. It has been suggested that IO leads to cardiomyopathy and heart failure [1,2,3], and its role in cardiac arrhythmogenesis remains controversial [4,5]

Recent studies have pointed towards IO as a potential contributor to mitochondrial dysfunction [6,7,8]. One mechanism by which Fe damages cardiomyocytes is thought to be mediated by excess reactive oxygen species (ROS) within mitochondria and/or the cytosol via the Fenton reaction [5,9]. A strong connection between ROS and cardiac diseases including heart failure, hypertrophy, and arrhythmias has been suggested [10,11,12,13]. Furthermore, mitochondria have been shown to play a vital role in cardiomyocyte intracellular Ca homeostasis [14,15,16]. 

Mitochondrial Ca efflux is primarily dependent on two mechanisms: the Na-Ca exchanger, which is the primary channel under physiologic conditions, and the mitochondrial permeability transition pore (mPTP) which opens during times of pathophysiologic stress [17]. These Ca channels/ transporters get their driving force from the Ca gradient and mitochondrial membrane potential (*ΔΨ_m_*) established by the proton gradient generated by the mitochondrial electron transport chain.

It has also been shown that mitochondria are physically associated with the sarcoplasmic reticulum (SR) [18]. This link is approximately 10 to 50 nm wide and may thus create micro-domains in which the intracellular Ca levels can be readily manipulated to induce a variety of ionic fluxes. Our previous study suggests that mitochondrial Ca efflux creates a micro-domain with an increase in local Ca between the ryanodine receptors and the SR Ca ATPase uptake channels, thereby altering the Ca handling properties of the SR and in the cytosol [19]. We have shown that the protonophore carbonyl cyanide *p*-(trifluoromethoxy) phenylhydrazone (FCCP) leads to *ΔΨ_m_* depolarization and mPTP opening, which subsequently allows for mitochondrial Ca release via the mPTP. The release of mitochondrial Ca promotes spontaneous Ca release from the SR, resulting in Ca waves (CaWs), which eventually promotes the generation of triggered electrical activities and arrhythmogenesis [19]. More recently, by using cyclosporin A (CsA), a selective inhibitor of mPTP, or the cyclophilin D knockout (CypD KO) mouse model, we have further demonstrated that inhibition of mPTP can attenuate FCCP-induced mitochondrial Ca efflux and subsequently prevent the occurrence of CaWs in ventricular myocytes as well as reduce the incidence of arrhythmias at the whole heart level [20]. However, a link between IO, mitochondrial mPTP activity, and cytosolic Ca mobilization has not been demonstrated to date. In the present study, we demonstrate that IO promotes ROS production, resulting in *ΔΨ_m_* depolarization. As a result, there is mitochondrial Ca efflux via mPTP which ultimately promotes CaW formation and arrhythmogenesis. For our studies, we have used both pharmacological (CsA) and genetic (CypD KO) approaches to inhibiting mPTP function.

## 2. Materials and Methods

### 2.1. Animal Models

As we described in our previous study [20], WT and CypD KO [21] mice (2–4 months, either gender) were purchased from The Jackson Laboratories (Bar Harbor, ME, USA). All animal experimental protocols were reviewed by the Institutional Animal Care and Use Committee at Rutgers New Jersey Medical School and were in accordance with the Guide for the Care and Use of Laboratory Animals published by the National Institutes of Health (Revised 1996, The Animal Welfare Assurance Number D16-00098; IACUC Protocol #: PROTO999901063).

### 2.2. Chemicals and Reagents

Most chemicals and reagents were purchased from Sigma-Aldrich (St. Louis, MO, USA) or Invitrogen by Thermo Fisher Scientific (Grand Island, NY, USA), as indicated. Hydrophobic reagents were dissolved in DMSO and then diluted to working concentrations in normal Tyrode’s solution. The maximum DMSO concentration was <0.2% by volume. A membrane permeable complex of Fe^3+^ and 8-hydroxyquinoline (8-HQ, Sigma-Aldrich) was used to iron load ventricular myocytes. It has been reported that, after the lipophilic Fe^3+^/8-HQ complex enters into the cell, Fe^3+^ ions undergo rapid intracellular reduction to Fe^2+^ [22]. Cytosolic Fe^3+^ and Fe^2+^ then become part of the pool of labile Fe within the cell, i.e., chelatable and redox-active [23].

### 2.3. Cell Isolation

Left ventricular myocytes were enzymatically isolated from mouse hearts, as described previously [24,25,26]. Briefly, the hearts were removed from mice deeply anesthetized with isoflurane (Henry Schein Animal Health, Dublin, OH, USA) and were retrogradely perfused at 37 °C in Langendorff fashion with nominally Ca-free Tyrode’s solution containing 0.5 mg/mL collagenase (Type II; Worthington Biochemical Co., Lakewood, NJ, USA) and 0.1 mg/mL thermolysin (Sigma-Aldrich) for 10–12 min. The enzyme solution was then washed out and the hearts were removed from the perfusion apparatus. The left ventricle was removed and placed in a petri dish. Myocytes were isolated after being teased apart using forceps and being filtered through a nylon mesh. The Ca concentration was gradually increased to 1.0 mM and the cells were stored at room temperature. Ventricular myocytes were studied within 8 h of isolation. 

### 2.4. Measurement of Cytosolic and Mitochondrial Fe Loading

To determine cytosolic ferrous iron (Fe^2+^) loading, ventricular myocytes were loaded with 40 µM Phen Green SK (Invitrogen) for 10 min at room temperature. In order to determine mitochondrial Fe^2+^, ventricular myocytes were loaded with 5 µM rhodamine B-[(1,10-phenanthroline-5-yl)-aminocarbonyl]benzyl ester (RPA; Squarix Biotechnology by Axxora, Recklinghausen, Germany) for 20 min at 37 °C. Fluorescence (Ex/Em: 484/520 nm for Phen Green and 543/560 nm for RPA) was monitored using an Eclipse TE200 inverted microscope (Nikon, Tokyo, Japan) and recorded using an Ixon Charge-Coupled Device (CCD) camera (Andor Technology, Concord, MA, USA).

### 2.5. Measurement of Mitochondrial Reactive Oxygen Species Levels

Ventricular myocytes were loaded with 5 µM MitoSOX Red (Invitrogen) for 30 min at 37 °C in order to visualize mitochondrial superoxide production. The fluorescence (Ex/Em: 485/585 nm) was monitored and presented as background-subtracted *F/F_0_* values. The *F/F_0_* value was expressed as “0” during the periods when the cell was not exposed to the excitation light. The baseline value (before perfusion of Fe^3+^/8-HQ) was normalized to 1. The results were obtained every 2 min by averaging 3 consecutive 200-ms exposures. 

### 2.6. Measurement of Mitochondrial Membrane Potential

Isolated ventricular myocytes were loaded with 50 nM tetramethylrhodamine methyl ester (TMRM, Invitrogen) at 37 °C for 40 min. Fluorescence (Ex/Em: 548/570 nM) was monitored and stored. A decrease in fluorescence rate was used as an index of mitochondrial membrane depolarization [19]. 

### 2.7. Measurements of Intracellular Ca Fluorescence

Ventricular myocytes were loaded with Fluo-4-AM (4 µM) (Invitrogen) for 40 min at room temperature, after which the incubating solution was removed and fresh Tyrode’s solution was added. Fluorescence (Ex/Em: 485/530 nm) was monitored as described in our previous studies [11,27]. Ca fluorescence intensity was recorded as the ratio *F/F_0_* (fluorescence (*F*) over the basal diastolic fluorescence (*F_0_*)).

### 2.8. Electrocardiograms (ECG) and Arrhythmia Induction Testing

Electrocardiograms (ECGs) were recorded at a sampling rate of 1 kHz in Langendorff-perfused hearts by placing an Ag-AgCl electrode pair close to the apex and on the right atrial appendage to produce a pseudo-lead II. Additionally, two platinum electrodes were placed on the right ventricular free wall for electrical stimulation (Grass Instruments, West Warwick, RI, USA). The hearts were perfused with normal Tyrode’s solution at 37 °C. The S1-S2 programmed electrical stimulation protocol (S1 being the regular train pulse and S2 the premature stimulus) at twice the pacing threshold intensity was used [20,26]. After a 20-beat train with 100-ms cycle length (S1), 3 repeated sets of 3 extra stimuli (S2) with cycle lengths of 50, 40, and 30 ms were introduced. Arrhythmia scores were assigned as follows: 0, no arrhythmia; 1 point, 1–3 premature ventricular contractions (PVCs); 2 points, non-sustained ventricular tachycardia (VT) (4–10 consecutive PVCs, including bigeminal or trigeminal PVCs); 3 points, sustained VT (>10 consecutive PVCs); 4 points, ventricular fibrillation (VF) or sudden cardiac arrest (SCD).

### 2.9. Statistics

Individual groups were compared by Student’s *t*-tests or Fischer’s exact tests, as indicated in the text. Results were considered statistically significant if the *p* value was less than 0.05. Results were expressed as mean ± SEM. 

## 3. Results

### 3.1. Fe Load in the Cytosol and Mitochondria in WT and CypD KO Ventricular Myocytes

**Figure 1:**
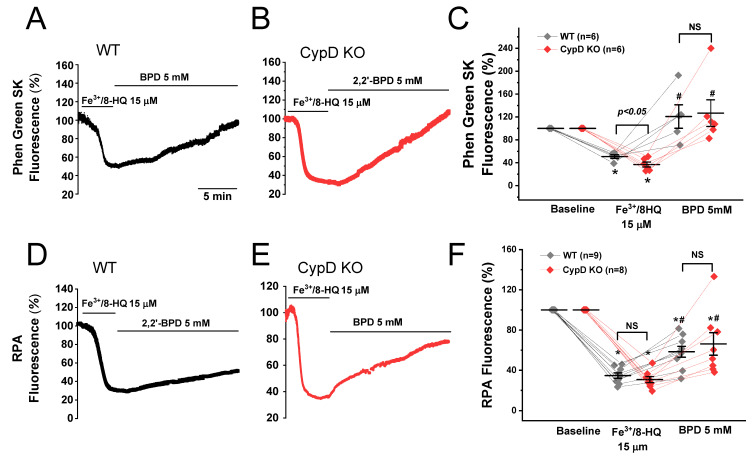
Cytosolic and mitochondrial Fe loading in ventricular myocytes isolated from WT and CypD KO mice. The level of cytosolic Fe was measured using Phen Green SK fluorescence in WT (**A**) and CypD KO myocytes (**B**). The baseline fluorescence levels (arbitrary units, AU) were normalized to 100%. Cytosolic Fe loading was achieved by continuous superfusion with 15 μM Fe^3+^/8-HQ. Decreased fluorescence intensity of Phen Green SK indicated the increase in cytosolic Fe levels. Note that the quenched fluorescence was reversed in the presence of the membrane-permeable Fe chelator 2,2′-bipyridyl (BPD). Summarized data (**C**) were obtained from 6 WT cells and 6 CypD KO cells, respectively. Similarly, mitochondrial Fe loading was measured by using rhodamine B-[(1,10-phenanthroline-5-yl)-aminocarbonyl]benzyl ester (RPA) fluorescence in WT (**D**) and CypD KO myocytes (**E**). Data are summarized in (**F**). Data were obtained from 9 WT cells and 8 CypD KO ventricular myocytes. * *p* < 0.05 compared to the baseline in WT and CypD, respectively; # *p* < 0.05 compared to the Fe^3+^/8-HQ treatment group in WT and CypD, respectively, by using paired *t*-test. Except for Fe^3+^/8-HQ-induced Phen Green SK fluorescence, no significant difference was observed between WT and CypD KO.

In order to establish IO, we mixed Fe^3+^ with the cell permeable molecule 8-hydroxyquinoline to form a Fe^3+^/8-HQ complex. To confirm that Fe^3+^/8-HQ is capable of readily entering the cell and that Fe^3+^ is reduced to Fe^2+^, we used Phen Green SK to visualize cytosolic Fe^2+^ levels and RPA to visualize mitochondrial Fe^2+^ levels. The baseline fluorescence levels (arbitrary units, AU) were normalized to 100%. As shown in Figure 1A,C, in WT myocytes, the treatment with Fe^3+^/8-HQ resulted in a rapid decrease (quenching) in Phen Green SK fluorescence (54.4 ± 4.5%), indicating that cytosolic Fe^2+^ was increased. Upon addition of the cell-permeable Fe chelator 2,2′-bipyridyl (BPD, 5 mM), fluorescence was restored to 118.4 ± 16.9% after 15 min (Figure 1A,C), indicating that labile Fe in the cytosol was successfully chelated by BPD, which is a strong cytosolic Fe chelator. The same experiments were conducted in ventricular myocytes isolated from CypD KO mice. As shown in Figure 1B,C, the treatment with Fe^3+^/8-HQ resulted in a significant decrease in Phen Green SK fluorescence to 36.9 ± 7.2%, which was recovered to 126.7 ± 23.3% after chelation with BPD. To determine whether mitochondrial Fe^2+^ level was elevated as well after Fe^3+^/8-HQ treatment, we monitored the fluorescence of RPA that is considered to be specific to mitochondrial Fe^2+^. As seen in Figure 1D,F, the average fluorescence of RPA in WT myocytes was significantly decreased to 34.8 ± 2.7% when the cells were perfused with 15 µM Fe^3+^/8-HQ, suggesting that mitochondrial Fe^2+^ was increased. BPD has also been reported to be a mitochondria-accessible Fe chelator [28]. When treated with BPD, a partial recovery of RPA fluorescence was observed (58.4 ± 5.4%), suggesting partial chelation of mitochondrial Fe^2+^. The same experiments were conducted in myocytes isolated from CypD KO mice. As shown in Figure 1E,F, the average fluorescence of RPA in CypD KO myocytes was not different from WT at baseline. After treatment with 15 µM Fe^3+^/8-HQ, RPA fluorescence was significantly decreased to 30.7 ± 3.0% in CypD KO, which was recovered to 66.2 ± 11.2% with the addition of BPD. These results clearly show that Fe^3+^/8-HQ treatment generates an IO condition in both WT and CypD ventricular myocytes, resulting in an increase in both cytosolic and mitochondrial Fe^2+^ levels. 

### 3.2. IO induced Mitochondrial ROS Generation and ΔΨ_m_ Depolarization

**Figure 2:**
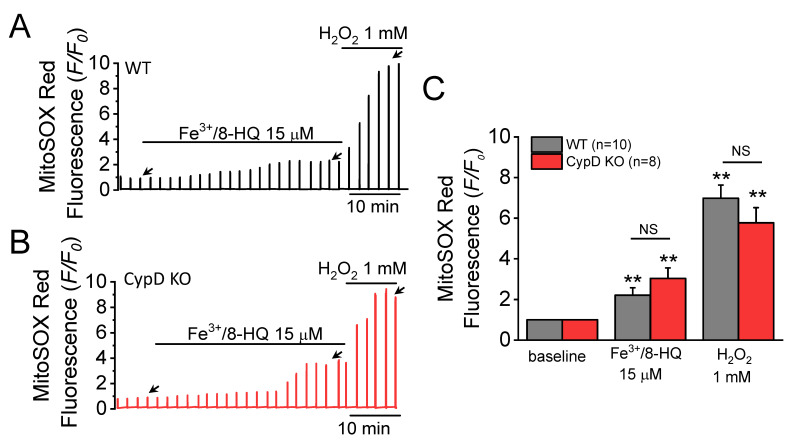
Fe-induced reactive oxygen species (ROS) generation in ventricular myocytes isolated from WT and CypD KO mice. Increases in MitoSOX Red fluorescence were used as an indicator of mitochondrial superoxide production. MitoSOX Red fluorescence traces were recorded in a WT (**A**) and a CypD KO myocyte (**B**) treated with 15 µM Fe/8-HQ. The baseline value was normalized to 1, as indicated by the left-most arrow in each panel. The effect of Fe treatment was measured at the point indicated by the middle arrow. H_2_O_2_ (1 mM) was used as a positive control indicator. Summary data (**C**) were obtained from 10 WT and 8 CypD KO myocytes, ** *p* < 0.01 compared to baseline in WT and CypD KO group, respectively, by using paired *t*-test. NS: no significant difference was observed between WT and CypD KO groups by unpaired *t*-test.

We next sought to evaluate mitochondrial ROS production in ventricular myocytes in the presence of IO. Perfusion with Fe^3+^/8-HQ (15 µM) significantly increased the fluorescence of MitoSOX Red, a mitochondrial ROS sensitive dye (Figure 2A–C). In both WT (Figure 2A) and CypD KO mouse myocytes (Figure 2B), fluorescence was increased, indicating that IO significantly increases mitochondrial ROS production. No significant difference was found between WT and CypD KO ventricular myocytes. H_2_O_2_ was used as a positive control following addition of 15 µM Fe^3+^/8-HQ. Addition of 1 mM H_2_O_2_ resulted in a further increase in mitochondrial ROS levels, potentially indicating that the relative maximum level of mitochondrial ROS generation was attained.

**Figure 3:**
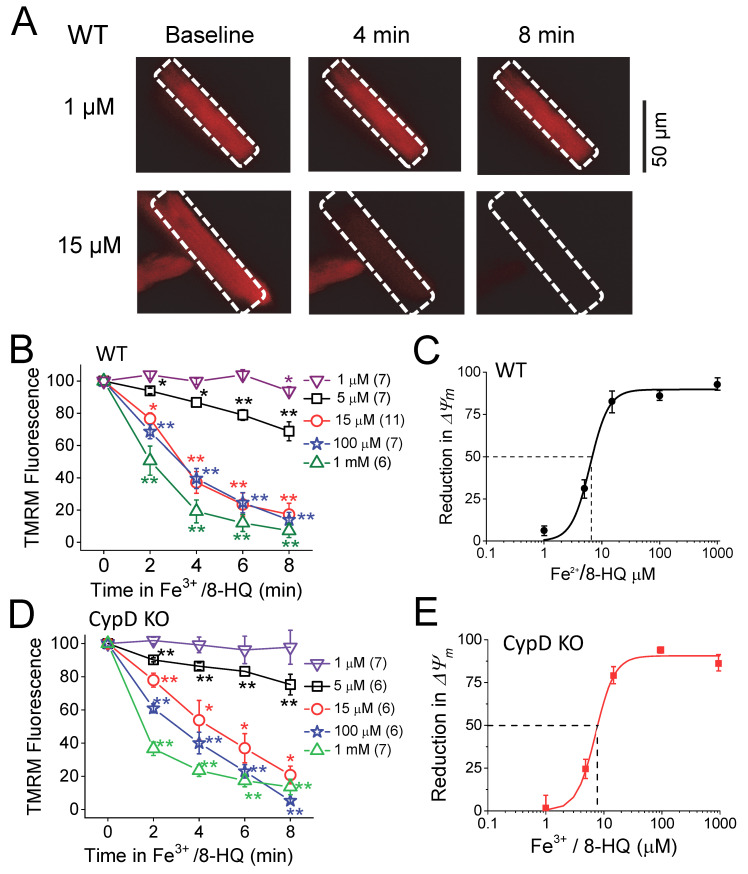
Fe-induced depolarization of mitochondrial membrane potential (*Δψ_m_*) in ventricular myocytes isolated from WT and CypD KO mice. (**A**) Representative snapshots of WT myocytes loaded with TMRM at baseline and 4 and 8 min after being treated with 1 and 15 µM Fe^3+^/8-HQ. (**B**) Summarized data showing a decrease in tetramethylrhodamine methyl ester (TMRM) fluorescence over time after Fe treatment in WT. The numbers of myocytes used in the measurement are indicated. (**C**) Percentile depolarization of *ΔΨ_m_* at 8 min after Fe treatment in WT fit to Hill equation: *ΔΨ_m_* = *ΔΨ_m,_*_max_*[Fe]^n_H_/(EC_50_^ n_H_ + [Fe]^n_H_). Half maximal effective dose (EC_50_) and Hill coefficient (n_H_) are 6.3 ± 0.3 µM and 2.7 ± 0.2, respectively, for WT. (**D**,**E**) In CypD KO myocytes, same as (**B** and **C**). EC_50_ and n_H_ are 7.2 ± 0.4 and 2.7 ± 0.1 for CypD KO. * *p* < 0.05, ** *p* < 0.01 compared to the respective baseline value. Carbonyl cyanide *p*-(trifluoromethoxy) phenylhydrazone (FCCP, 30 µM) was used to completely dissipate the *ΔΨ_m_* after the Fe treatment in each recording (not shown).

Next, we assessed *ΔΨ_m_*. As shown in Figure 3, *ΔΨ_m_* was monitored by TMRM fluorescence in WT (Figure 3A–C) and CypD KO (Figure 3D,E) myocytes. Myocyte membrane potential was depolarized with the addition of Fe^3+^/8-HQ in a dose- and time-dependent manner in both WT and CypD KO myocytes. While Fe^3^^+^/8-HQ at a low concentration (1 μM) caused less depolarization, 15 μM Fe^3+^/8-HQ induced significant depolarization starting at 2 min and reached quasi-maximal effect at 8 min after treatment. We fitted data at the eight-minute time point to the Hill equation (see figure legend for details). As shown in Figure 3C, the half maximal effective concentration (EC_50_) was 6.3 ± 0.3 µM of Fe^3+^/8-HQ for depolarization of the *ΔΨ_m_*, while the Hill coefficient (n_H_) was 2.7 ± 0.2 in WT myocytes. In CypD KO myocytes (Figure 3D,E), we obtained an EC_50_ of 7.2 ± 0.4 µM and n_H_ of 2.7 ± 0.1, which were not significantly different from WT. These results suggest that IO may cause the same level of depolarization of mitochondrial *ΔΨ_m_* in both WT and CypD KO myocytes. 

### 3.3. Promotion of Ca Waves by IO and Protection by mPTP Inhibition

**Figure 4:**
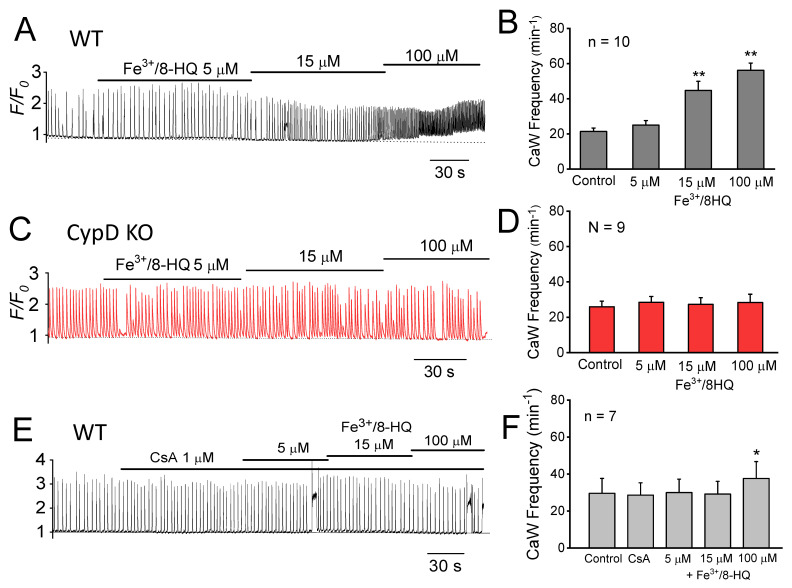
Promotion of Ca wave generation by Fe and attenuation by mitochondrial permeability transition pore (mPTP) Inhibition. (**A**) A representative Ca fluorescence trace showing the effect of Fe^3+^/HQ-8 (5, 15, and 100 µM) on the frequency of spontaneous Ca wave formation in a WT ventricular myocyte. (**B**) Summarized data obtained from 10 WT myocytes. (**C**,**D**) The same as (**A**,**B**), except in CypD KO ventricular myocytes (*n* = 9). (**E**,**F**) The same as (**A**,**B**), except that the WT myocytes were pretreated with 1 µM CsA before Fe perfusion (*n* = 7). * *p* < 0.05, ** *p* < 0.01, compared to respective control value by paired *t*-test.

In a previous study, we demonstrated that depolarization of *ΔΨ_m_* (by FCCP) promotes CaW generation, presumably by opening mPTP that releases mitochondrial Ca and triggers SR Ca release [19,20]. We next tested whether IO might also accelerate CaW formation via mPTP activation. As shown in Figure 4A,B, Fe^3+^/8-HQ significantly increased the frequency of CaW formation in a dose-dependent manner in WT ventricular myocytes. The frequency of formation of CaWs increased from 21.4 ± 1.9 to 44.7 ± 5.3 min^−1^ (*p* < 0.01) at 15 µM, while it was further increased to 56.2 ± 4.1 min^−1^ (*p* < 0.01) at 100 µM. To determine the role of mPTP, we studied ventricular myocytes from CypD KO mouse hearts which have impaired mPTP function [21]. As shown in Figure 4C,D, CypD KO myocytes perfused with the same concentrations of Fe^3+^/8-HQ showed no significant increase in the frequency of formation of CaWs (from baseline 26.0 ± 3.2 min^−1^ to 27.3 ± 3.8 min^−1^ at 15 µM and 28.4 ± 4.6 min^−1^ at 100 µM, *p* > 0.05). The dose-dependent increase in the frequency of CaW formation in WT myocytes was abrogated by pretreatment with CsA, a selective mPTP blocker. As shown in Figure 4E,F, Fe^3+^/8-HQ did not cause a significant increase in the frequency of CaW formation in the presence of 1 µM CsA (28.6 ± 6.7 min^−1^ to 30.0 ± 7.2 min^−1^ at 5 µM, and 29.1 ± 6.9 min^−1^ at 15 µM, *p* > 0.05). However, 1 µM CsA did not completely abolish the effect of Fe^3+^/8-HQ at the higher concentration of Fe^3+^/8-HQ (100 µM). Fe^3+^/8-HQ at 100 µM resulted in a slight but significant increase in the frequency of CaW formation (28.6 ± 6.7 min^−1^ vs 37.6 ± 9.2 min^−1^, *p* < 0.05). Taken together, these results suggest that mPTP-mediated Ca release from mitochondria may contribute to IO-induced CaW formation. Inhibition of mPTP function by either pharmacologic (CsA) or genetic (CypD KO) approaches attenuated CaW formation. 

### 3.4. Preventative Effect of Antioxidants against IO Toxicity 

**Figure 5:**
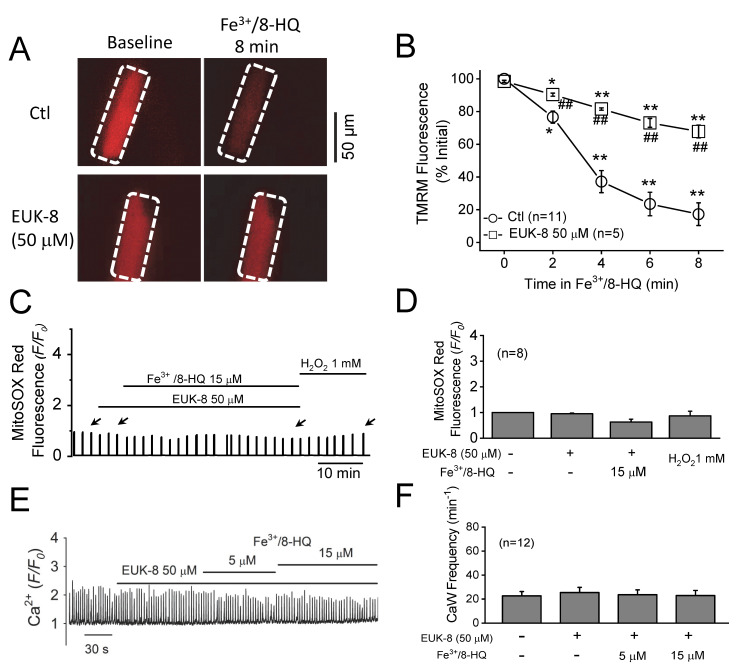
Attenuation of Fe-induced mitochondrial dysfunction and Ca waves by antioxidants. (**A**) Representative snapshots of WT myocytes loaded with TMRM at baseline and 8 min after being treated with 15 µM Fe^3+^/8-HQ in the absence or presence of 50 µM EUK-8. Arrows indicate the time points where fluorescence values were measured. (**B**) Summarized data showing a decrease in TMRM fluorescence over time after Fe treatment in WT (*n* = 11). * *p* < 0.05, ** *p* < 0.01, compared to baseline, respectively. ^##^ *p* < 0.01 compared between control and EUK-8 groups (*n* = 5). (**C**) A representative MitoSOX Red fluorescence trace recorded in a WT treated myocyte with 15 µM Fe/8-HQ in the presence of EUK-8. (**D**) Summarized data of MitoSOX Red fluorescence recorded from 8 WT myocytes. (**E**) A representative CaW trace showing the effect of pretreatment with EUK-8 on the frequency of Fe-induced CaW formation in a WT ventricular myocyte. (**F**) Summarized data of the frequency of CaW formation recorded from 12 myocytes.

To further examine the contribution of an increase in the production of ROS to CaW formation resulting from IO, we tested the possible preventative effect of EUK-8, a strong synthetic antioxidant that exhibits both superoxide dismutase and catalase activities. As shown in Figure 5A,B, we pretreated ventricular myocytes with 50 µM EUK-8 for 2 min, followed by concurrent perfusion with Fe^3+^/8-HQ for eight minutes. Although Fe^3+^/8-HQ treatment still depolarized *ΔΨ_m_* (decrease in TMRM fluorescence) in the presence of 50 µM EUK-8, the extent of depolarization was weaker than what we observed in the control group. Furthermore, treatment with Fe^3+^/8-HQ (15 µM) resulted in no significant increase in mitochondrial superoxide generation (as monitored by MitoSOX Red) in the presence of EUK-8 (Figure 5C,D). The effect of subsequent treatment with exogenous H_2_O_2_ (1 mM) was also attenuated by EUK-8. More importantly, as shown in Figure 5E,F, pretreatment with EUK-8 prevented an increase in the frequency of CaW formation seen with 15 µM Fe^3+^/8-HQ. Similar results with regard to ROS and CaW formation were also observed in cells pretreated with 20 µM MitoTEMPO (a mitochondrial-targeted superoxide scavenger) (data not shown). These results clearly reveal that antioxidants can attenuate IO-induced mitochondrial ROS production, *ΔΨ_m_* depolarization, and CaW formation. 

### 3.5. IO Promoted Arrhythmias and Their Prevention by Antioxidants and mPTP Inhibition in Ex-Vivo Hearts

**Figure 6:**
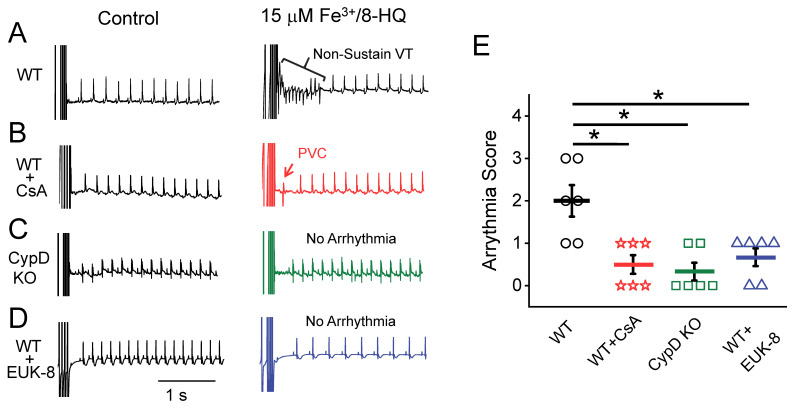
Fe-induced arrhythmias were attenuated by antioxidants and mPTP inhibition in ex-vivo mouse hearts. Pseudo-lead II ECG signals recorded from ex-vivo mouse hearts at baseline and after treatment with 15 µM Fe/8-HQ in the presence/absence of other agents. S1-S2 programmed stimulations (as indicated by the stimulation artifacts at the beginning of each trace) were applied. (**A**) WT mouse heart treated with 15 µM Fe^3+^/8-HQ. (**B**) WT mouse heart treated with 15 µM Fe^3+^/8-HQ in the presence of 1 µM CsA. (**C**) CypD KO mouse heart treated with 15 µM Fe^3+^/8-HQ. (**D**) WT mouse heart treated with 15 µM Fe^3+^/8-HQ in the presence of 25µM EUK-8. (**E**). Summarized arrhythmia scores (*n* = 6 in each group). See Methods for details. * *p* < 0.05 compared to Fe only treatment group in WT.

Cytosolic CaWs have been implicated in the generation of arrhythmias in the heart. We next evaluated the arrhythmogenic effect of IO using an ex-vivo whole-heart preparation. Representative pseudo-lead II ECGs were recorded from Langendorff-perfused hearts with normal Tyrode’s solution containing 15 μM Fe^3+^/8-HQ. The susceptibility to S1-S2 stimulation-induced arrhythmias was evaluated. As shown in Figure 6A, while control hearts (vehicle group with 15 μM 8-HQ) showed no arrhythmias, an episode of non-sustained VT was evident in the 15 μM Fe^3+^/8-HQ perfusion group. In another group, WT hearts were pretreated with the mPTP inhibitor CsA (1 µM) and then concurrently perfused with 15 µM Fe^3+^/8-HQ. The hearts in this group were significantly protected from arrhythmogenesis by IO (Figure 6B), with only occasional PVCs being observed. Similar to findings with CsA, in CypD KO mouse hearts, there was less induction of arrhythmias (Figure 6C). Furthermore, the proarrhythmic effect of IO was also attenuated by pretreatment with the antioxidant EUK-8 that exhibits both superoxide dismutase and catalase activities (Figure 6D). As summarized in Figure 6E, WT hearts were particularly susceptible to arrhythmogenesis, represented by sustained VT, non-sustained VT, and PVCs, and had an arrhythmia score averaging 2.00 ± 0.37. Significantly lower arrhythmia scores were obtained with CsA (0.50 ± 0.22) and EUK-8 (0.67 ± 0.21) pretreatment and in the CypD KO group (0.33 ± 0.21). 

## 4. Discussion

Recent studies have revealed that IO may induce mitochondrial dysfunction [6,7,8]. To determine the potential link between IO and its effect on mitochondrial function and cytosolic Ca handling, we used the Fe^3+^/8-HQ complex to iron load both cytosol and mitochondria. We have found that Fe treatment results in excess ROS generation, *ΔΨ_m_* depolarization, and an increase in cytosolic CaW frequency. In agreement with the notion that cardiac arrhythmias are associated with CaWs, we have also demonstrated that IO increases the incidence of arrhythmias in ex-vivo hearts with a S1-S2 stimulation protocol. After pretreatment with CsA or in ventricular myocytes isolated from CypD KO mice, the effects of IO were alleviated, implicating an essential role of mPTP that links IO-induced *ΔΨ_m_* depolarization to mitochondrial Ca efflux and CaWs/arrhythmias. Our study has not only a strong basic science component that enables us to better understand the impact of IO on mitochondrial function and Ca handing, but it also has high translational impact. We have tested possible therapeutic approaches that might address important clinical questions related to IO-induced cardiomyopathy and cardiac arrhythmias.

### 4.1. Iron Overload and Arrhythmogenesis—Discrepancies in Clinical and Experimental Settings

While it has been well acknowledged that IO results in cardiomyopathy [1,2,3], it remains unclear whether IO may play a causal role in cardiac arrhythmogenesis and how mitochondrial dysfunction might be involved [4,5]. Clinical studies have reported the occurrence of atrial and ventricular tachyarrhythmias in patients with IO conditions [4,29,30,31,32,33,34]. For example, sustained ventricular tachycardia was observed in thalassemic patients with IO [29] Furthermore, Mancuso et al. found low voltages in ECG recordings, T wave inversions, and supraventricular arrhythmias in thalassemic patients suffering from heart failure, while their age and sex matched controls had no ECG abnormalities [30]. In addition, O in patients with sickle cell disease and hereditary hemochromatosis has been associated with the incidence of sudden cardiac death induced by severe arrhythmias [35,36]. Furthermore, Fe deposition may occur in the entire cardiac conduction system, especially the atrioventricular node, resulting in first-degree, second-degree, and complete atrioventricular block [37]. 

Several experimental studies have reported that chronic IO results in arrhythmias and cardiomyopathy in animal models [38,39]. For example, chronic IO has been demonstrated to result in prolonged PR intervals, heart block, and atrial fibrillation in a mouse model [38]. In addition, abnormal ECGs were present in gerbils after IO, which demonstrated prolongation of the QRS complex and PR intervals, PVCs, atrioventricular block, ST segment elevation, and T-wave inversion [39]. However, studies by Kaiser et al. have claimed that guinea pigs [40] and gerbils [40,41] do not display arrhythmias, despite showing hallmark symptoms of IO such as significant increases in cardiac and hepatic Fe deposition and cardiac and liver fibrosis. While these results imply the absence of a causal link between IO and arrhythmogenesis, their conclusions were based on spontaneously occuring arrhythmic events. The authors did not attempt to examine the effect of IO under stressed conditions such as arrhythmia induction testing, simulated hypercalcemia, or sympathetic hyperactivity (β-adrenergic stimulation). 

In our present study, we have employed an ex-vivo cardiac model using programmed S1-S2 pacing protocols (similar to induction protocols used clinically to induce arrhythmias). While the occurrences of atrioventricular block were also noticed under IO conditions, we have focused on S1-S2 stimulation-facilitated ventricular tachyarrhythmias. We have found that IO increases the susceptibility to induced arrhythmias such as VTs in WT mouse hearts. In contrast, mPTP inhibition, either through pharmacological (CsA) or genetic intervention (CypD KO), significantly reduced the incidence of IO-induced ventricular tachyarrhythmias. These results suggest that acute IO may exert a proarrhythmic effect via *ΔΨ_m_* depolarization and increased activity of mPTP, resembling our previous findings with FCCP in ventricular myocytes [19,20]. It should also be noted that IO per se may not be sufficient to readily cause arrhythmias, as other additive or synergistic factors may also be required. These factors may include elevated β-adrenergic stimulation, oxidative stress, and Ca overload as well as fast pacing (e.g., the programmed S1-S2 pacing as used in the present study). It remains to be further studied how other potential factors may work additively/synergistically in combination with IO conditions. 

Our present proof-of-concept study has demonstrated the importance of mitochondrial function and especially mPTP in iron overload-induced Ca dysregulation and arrhythmogenesis. We acknowledge that further in-vivo experiments should provide greater insights into the overall pathological effects of iron overload as well as Fe homeostasis while allowing identification of potential compensatory mechanism(s). Further future studies using a chronic in-vivo iron overload model are warranted.

### 4.2. Mechanisms for Ca Mishandling and Arrhythmias under IO: Roles of mPTP and Other Targets

Mitochondrial Fe is necessary for heme biosynthesis and iron-sulfur cluster biosynthesis, which are important for erythropoiesis and mitochondrial metabolism under normal physiological conditions [42]. However, excess Fe in mitochondria leads to dysfunction of these important homeostatic activities. As we discussed in our recent review article [5], it seems that IO in mitochondria plays a critical role in causing cellular oxidative stress, mitochondrial dysfunction, as well as cardiomyopathy and potentially arrhythmias. Mitochondria are located in close proximity to the SR, as well as to the calcium-releasing ryanodine receptors. This micro-domain has recently been shown to contain gradients in Ca levels [43], thus suggesting a functional relationship that involves ion fluxes between the two organelles. Our previous studies have demonstrated that FCCP-induced mitochondrial dysfunction (i.e., *ΔΨ_m_* depolarization) leads to mPTP opening that allows for mitochondrial Ca release and exacerbates CaW formation. This eventually promotes the generation of triggered activities and arrhythmias [19,20]. Since IO also induces *∆Ψ_m_* depolarization, we postulate that IO could similarly promote CaWs and arrhythmias via the same mechanistic link, i.e., mPTP opening, release of Ca from mitochondria, and triggering of frequent SR Ca release (i.e., CaW formation). This postulation is supported by the evidence that inhibition of mPTP (CsA or CypD KO) attenuates IO-promoted CaWs in myocytes and arrhythmias in ex-vivo hearts.

It has been revealed that mitoferrin-1 and 2 (Mfrn1 and 2) are localized on the inner membrane of mitochondria and may play an important role in Fe uptake into the mitochondria [42,44,45]. Furthermore, a very recent report has proposed that the mitochondrial calcium uniporter (mCU) may also transport Fe into mitochondria, while Mfrn2 serves to regulate mCU transport [46]. Interestingly, some recent studies have suggested that mCUs are also involved in brain and heart mitochondrial dysfunction under IO conditions [7,29]. As summarized in our review article [47], Fe also exerts effects on ion channels in cardiomyocytes, e.g., L- and T-type Ca channels, K and Na channels. Recently, we have discovered that Fe also activates transient receptor potential canonical (TRPC) channels, which results in alterations in membrane potential, Ca handling, and cardiac dysfunction [48]. In addition, IO-enhanced ROS generation may activate calcium/calmodulin-dependent protein kinase II via oxidation and subsequently affect many ionic channels/transporters, e.g., I_Na_, I_CaL_, and RyR, and promote afterdepolarizations [11,49,50,51]. Therefore, it is conceivable that IO-induced cardiac dysfunction/arrhythmias may be mediated by multiple targets including ROS generation, mitochondrial function, mitochondrial and SR Ca handling, as well as sarcolemmal ion channels. A comprehensive approach may be necessary for effective prevention/treatment for IO-associated cardiac disease.

### 4.3. IO-Induced Oxidative Stress and the Effect of Antioxidants

In agreement with the notion that Fe participates in the Fenton reactions generating free radicals, we observed a significant increase in ROS levels in mitochondria after Fe treatment. We also found that Fe caused *ΔΨ_m_* depolarization in a dose-dependent manner in both WT and CypD KO. Since mPTPs are Ca, redox, voltage, and pH sensitive, their opening is promoted by free Ca in the mitochondrial matrix, ROS, *ΔΨ_m_* depolarization, and an alkaline pH [52]. It has been well established that the mPTP can open in response to mitochondrial stress, including a buildup of reactive oxygen species [53,54], and ischemia-reperfusion injury [55]. It has been suggested that formation of disulfide bonds between critical thiol groups on the adenine nucleotide translocator (ANT) may facilitate CypD binding to promote mPTP opening, which may be the basis for the effects of ROS [56]. In our previous studies, we used the protonophore FCCP to dissipate the proton gradient and to directly depolarize *ΔΨ_m_* in order to cause mPTP opening [19,20]. In the present study, both IO-promoted ROS generation and *ΔΨ_m_* depolarization should increase the probability of mPTP opening, while CypD KO should attenuate the probability of mPTP opening. Our present results are consistent with the findings of Sripetchwandee et al., who showed that Fe is capable of generating mitochondrial ROS that then depolarize the *ΔΨ_m_* and open the mPTP, resulting in mitochondrial swelling [7].

### 4.4. Limitations

The calcein/cobalt-quenching assay has been used by other investigators and ourselves to determine the extent of mPTP opening [19,57]. However, it should be noted that mPTP opening could not be evaluated by using this assay under IO conditions as calcein fluorescence is also quenched directly by Fe. Indirect evaluation of mPTP opening by measuring mitochondrial swelling [58] may serve as another method to determine the extent of mPTP opening. In fact, a recent study from our collaborator’s group has shown that exogenous Fe leads to mitochondrial swelling and mPTP opening [7]. In addition, evaluation of mitochondrial respiration using the Seahorse analyzer may also provide insights into changes in mitochondrial function under IO conditions [59]. Nevertheless, our experiments using the mPTP inhibitor CsA and CypD KO mice provide direct evidence of the role of mPTP under IO conditions. 

## 5. Conclusions

Our present study demonstrates that IO induces mitochondrial ROS generation and ∆*Ψ*_m_ depolarization, thereby opening the mPTP and promoting CaWs and cardiac arrhythmias. Conversely, the inhibition of mPTP ameliorates the proarrhythmic effects of IO.
